# Postnatal Hematopoiesis and Gut Microbiota in NOD Mice Deviate from C57BL/6 Mice

**DOI:** 10.1155/2016/6321980

**Published:** 2015-12-10

**Authors:** Dina Silke Malling Damlund, Stine Broeng Metzdorff, Jane Preuss Hasselby, Maria Wiese, Mia Lundsager, Dennis Sandris Nielsen, Karsten Stig Buschard, Axel Kornerup Hansen, Hanne Frøkiær

**Affiliations:** ^1^Department of Veterinary Disease Biology, Faculty of Health and Medical Sciences, University of Copenhagen, 1870 Frederiksberg C, Denmark; ^2^Department of Pathology, Rigshospitalet, 2100 Copenhagen, Denmark; ^3^Department of Food Science, University of Copenhagen, 1870 Frederiksberg C, Denmark; ^4^Bartholin Institute, Rigshospitalet, 2100 Copenhagen, Denmark

## Abstract

Neonatal studies in different mouse strains reveal that early life colonization affects the development of adaptive immunity in mice. The nonobese diabetic (NOD) mouse spontaneously develops autoimmune diabetes, but neonatal studies of NOD mice are lacking. We hypothesized that NOD mice deviate from another much used mouse strain, C57BL/6, with respect to postnatal microbiota and/or hematopoiesis and compared this in newborn mice of dams housed under the same conditions. A distinct bacteria profile rich in *staphylococci* was found at postnatal days (PND) 1–4 in NOD mice. Furthermore, a distinct splenic cell profile high in a granulocytic phenotype was evident in the neonatal NOD mice whereas neonatal C57BL/6 mice showed a profile rich in monocytes. Neonatal expression of *Reg3g* and *Muc2* in the gut was deviating in NOD mice and coincided with fewer bacteria attaching to the Mucosal surface in NOD compared to C57BL/6 mice.

## 1. Introduction

The nonobese diabetic (NOD) mouse spontaneously develops autoimmune diabetes and provides a suitable rodent model for research in type 1 diabetes pathogenesis [1, 2]. Host genetics, gut microbiota, and diet-associated factors are central in the development of diabetes in NOD mice [3–7]. However, the mechanisms behind the microbiota-host interaction and the specific genetic deviations leading to the disease pathology of type 1 diabetes are poorly understood.

The development of autoimmune diabetes in NOD mice is a result of polygenic interactions. The etiology is complex [5, 8], with genetics playing an important role, but also other factors influence the disease pathology [9]. The incidence of diabetes in NOD mice has been correlated with hygiene and microbiota in the colony and housing facility [4, 10] and gut microbiota (GM) dysbiosis have been linked to type 1 diabetes development [11, 12]. Factors determining GM composition are complex. Type of delivery and environmental factors have been implicated to play important roles in the establishment of GM. Children delivered by caesarean section may display an increased risk to develop autoimmune diabetes [13] indicating that early life colonization influences the development of type 1 diabetes.

Postnatal development of the immune system is heavily influenced by GM [14]. Germ-free mice have an underdeveloped mucosal epithelium with smaller Peyer's patches, fewer isolated lymphoid follicles, and reduced production of antimicrobial peptides [15]. GM composition is influenced by antimicrobial peptides [16] and one function of the mucus layer is to retain defensins and antimicrobial proteins, such as the antimicrobial C-type lectin Reg3g, in close proximity to the epithelial surface [17]. Reg3g is normally expressed from weaning and restricts the number of surface-associated bacteria [16], mainly targeting mucosa-associated Gram-positive bacteria [18]. In inflammatory bowel disease expression of the secreted mucus protein Muc2 is downregulated [19] while Reg3g expression is increased [20] indicating that Reg3g expression increases when the mucus layer is disassembled.

It has been suggested that genetic defects involved in the development of type 1 diabetes in NOD mice are linked to differentiation and proliferation of hematopoietic stem cells (HSCs) [21, 22]. Notably, bone marrow transplantation from young BALB/c mice to NOD mice prevents and reverses the development of autoimmune diabetes [21]. Langmuir et al. [23] demonstrated that expression of the hematopoietic differentiation antigen Ly6C, expressed on monocytes, neutrophils, and T cells [24, 25], is severely impaired in NOD mice. An interruption in the flanking region of the Ly6C gene upstream of the transcription initiation site is present in the NOD mice genome [26], which appears to hamper the transcription. The Ly6C gene is not completely knocked out in NOD mice, as some Ly6C expression is still detectable. Stimulation by cytokines does not restore the expression of the gene [26].

Knowledge regarding deviations in the development and differentiation of HSCs and defects in myelopoiesis as well as gut colonisation and gene expression here might aid to understand autoimmunity in NOD mice. We therefore compared development and activation of the myeloid cell lineage and GM composition in NOD and C57BL/6 mice focusing on the very first days after birth and found significant differences between the two strains, as regards both neonatal GM and hematopoiesis.

We speculate that NOD mice in the neonatal period have an altered GM, which in turn may influence the development of the genetically deviating immune system.

## 2. Experimental Procedures

### 2.1. Animals and Tissue

NOD/BomTac and C57BL/6 mice were purchased from Taconic (Hudson, NY, USA, and Denmark, resp.) and housed in a barrier protected rodent facility (Faculty of Health and Medical Science, University of Copenhagen, Frederiksberg, Denmark) under standard conditions in open cages without filter and with access to water and food (Altromin 1324, Lage, Germany)* ad libitum*. The two strains were mated separately and postnatal day 1 (PND 1) was defined as the day pups were born. 16–18 pubs from each strain were euthanized at PND 1, 2, 4, 7, 21, 28, and 70 by decapitation (PND 1–7) and cervical dislocation (PND 21–70). 6–8 spleens from each strain were dissected, kept on ice, and prepared for flow cytometry. 4–6 spleens, liver, and intestine samples were transferred to RNAlater (Ambion) for RNA and DNA extraction and further 2-3 spleens and livers were collected for histopathology examinations.

The project was licensed by the National Committee for Animal Experimentation under the Danish Ministry of Food, Fisheries and Agriculture (http://www.indberetning.dyreforsoegstilsynet.dk/) operating according to the EU directive 2010/63/EU (license number 2012-15-2934-00256-C1).

### 2.2. Splenic Cell Suspensions

Preparation of single cell suspensions was carried out by mechanical disruption of the spleen. Splenocytes were washed in ice cold sterile RPMI 1640 (Lonza), centrifuged for 10 min (200 g, 4°C). Erythrocytes were lysed by resuspension in 4 mL 0.2% sterile filtrated saline for 30 sec., following addition of 4 mL 1.6% saline. The procedure was repeated until the pellet was visibly free of erythrocytes. Cells were washed in RPMI, before viable cell count.

### 2.3. Analysis of Cell Population

Splenic single cell suspensions (1 × 10^5^–1 × 10^6^ cells/well) were incubated in the dark for 10 min with 3 *μ*g/mL anti-CD16/32 (BD Biosciences, Franklin Lakes, NJ, USA). Subsequently the following antibodies were added: anti-CD11b PE-cy7 clone M1/70, anti-Ly6C PE clone AL-21, and Ly6G APC clone 1A8 (eBiosciences, San Diego, CA, USA). Cells were analysed immediately on a FACScanto flow cytometer (BD Biosciences). Data analysis and layouts of FACS data were performed using Flowjo V 10.6 (Tree Star, Ashland, OR, USA).

### 2.4. RNA Isolation, cDNA Synthesis, and Gene Expression Analysis by qPCR

RNA extraction from samples stored in RNAlater and subsequently cDNA synthesis were performed as previously described [27].

Gene expression of* Reg3g*,* Camp*,* Arg1*,* Elane*,* Hp*,* Muc2,* and* Actb* was analysed as previously described [27] in samples from spleen, liver, and intestine using TaqMan Gene Expression Assays (Life Technology, Table 1). Fold changes in gene expression were calculated as 2^−ΔΔCt^ using the ΔΔCt method [28]. Briefly the expression of each sample was normalized to the expression of the reference (*Actb*) gene: (ΔCt = Ct(target) − Ct(reference)), and the relative gene expression was calculated as (ΔΔCt = ΔCt(target)  −  ΔCt(control)), where ΔCt(control) is the average ΔCt from NOD PND 1 samples.

### 2.5. MiSeq-Based 16S rRNA Gene Amplicon High Throughput Sequencing

31 mouse GM profiles were determined using tag-encoded 16S rRNA gene amplicon MiSeq (Illumina) based high throughput sequencing. DNA extraction and 16S rRNA gene library preparation were performed as previously described [29].

The raw dataset was trimmed using the “trim sequences” tool in CLC Genomic Workbench (CLC bio, Aarhus, Denmark). The fasta file format was made compatible with the Quantitative Insight Into Microbial Ecology (QIIME) open source software package (1.8.0) with an in-house Matlab script. Purging the dataset from chimeric reads and OTU clustering was conducted using UPARSE-. TU [30]. The relative distribution of the genera registered was calculated for unified and summarized in the genus level OTU tables. Alpha and beta-diversity measures, including analysis of group differences based on uniFrac distance metrics and ANOSIM analysis, were determined and analysed as previously described [29].

### 2.6. Generation and Harvest of Murine Bone Marrow-Derived Cells

Bone marrow cells were obtained from femur and tibia from adult NOD and C57BL/6 mice as previously described [31]. Cell suspensions were diluted to a concentration of 3 × 10^5^ cells/mL containing GM-CSF and incubated at 37° in a 5% CO_2_-incubator (day 0). On day 3 the cultured cells were stimulated with* Escherichia coli* Nissle 1917 (MOI 0.1),* Lactobacillus acidophilus* NCFM (MOI 0.1), or a mix of both (MOI 0.05 each). The cells were harvested at day 6, stained and analysed by flow cytometry.

### 2.7. Hematoxylin and Eosin (HE) Staining and Gram Staining

Spleens, liver, and intestine were formalin fixed and paraffin embedded, cut into 4 *μ*m thin sections, and mounted on superfrost plus slides and HE stained. The slides were evaluated by blinded visual inspection including an experienced pathologist. The degree of granulocytic infiltration was scored as 0 (no infiltration), 1 (mild focal infiltration), 2 (moderate focal infiltration), or 3 (severe infiltration). The proportion of hematopoietic tissue was scored using similar criteria.

Liver and spleen tissue were cut into 4 *μ*m thin sections and Gram-stained as described by Bancroft et al. [32].

### 2.8. Tissue Preparation and Projection Tomography (OPT) Scanning

To visualize the intestinal anatomy at PND 1 in NOD and C57BL/6 mice (two mice from each strain) the intestine was dissected and processed for optical projection tomography (OPT) as earlier described [33]. Each sample was scanned using the Biotonics 3001 OPT scanner (Bioptonics, Edinburgh, UK) at 425 nm with a rotation degree of 45° yielding 800 images with a resolution of 1024 × 1024 pixels. All samples were scanned at the same exposure time and same zoom factor. Scans were reconstructed with the NRecon V. 1.6.9.3 (Skyscan) software and digital sections illustrated in gray-scale demonstrating autofluorescence anatomy data were prepared with the Bioptonic Viewer Software V.2.0 (Bioptonics).

### 2.9. Statistical Analysis

For all statistical analysis, a two-way ANOVA test with Bonferroni post test was performed by Graph Pad Prism version 5.02 (Graphpad Software, San Diego, CA). In gene expression experiments, statistical analysis was performed on ΔCt values as these are assumed normally distributed as opposed to the fold change values. Statistical significance was accepted at ^*∗*^ *p* < 0.05, ^*∗∗*^ *p* < 0.01, and ^*∗∗∗*^ *p* < 0.001.

## 3. Results

### 3.1. Newborn NOD Mice Deviate in the Ly6G^+^/Ly6C^+^ Splenocyte Population

**Figure 1:**
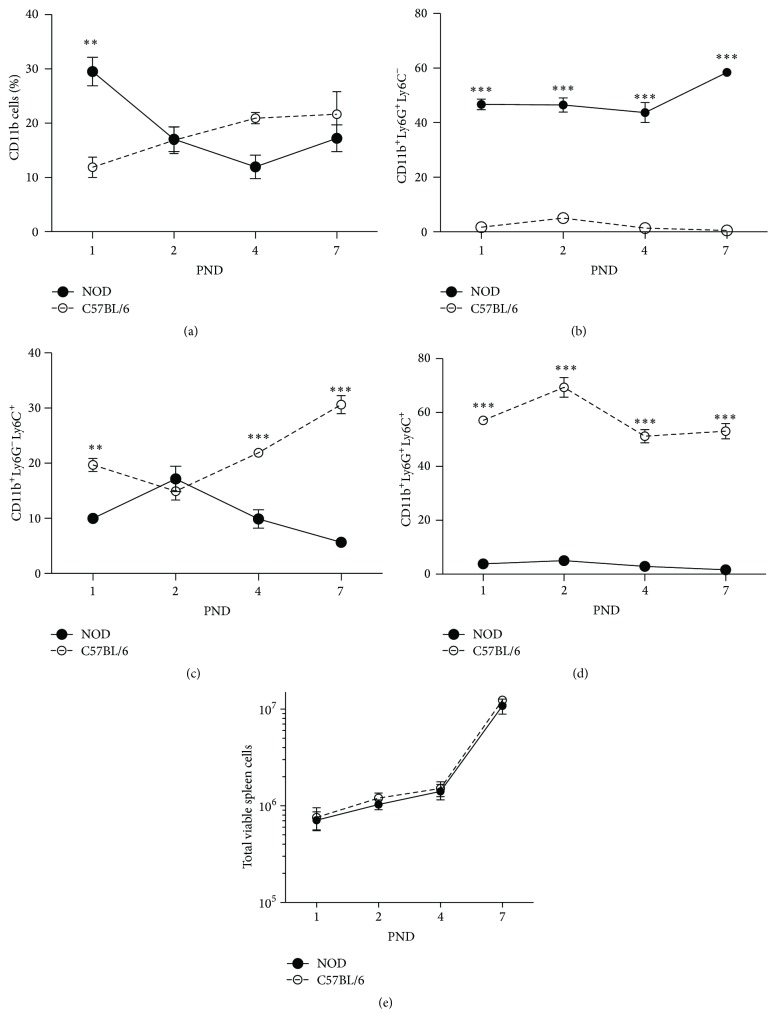
The proportion of CD11b^+^Gr-1^+^ splenocytes of newborn NOD mice is high compared to C57BL/6. Flow cytometric analysis of freshly isolated splenocytes from newborn NOD and C57BL/6 mice for the expression of CD11b, Ly6C, and Ly6G. (a) Time course development of the proportion of viable CD11b^+^ spleen cells in NOD and C57BL/6 mice at PND 1, 2, 4, and 7. Graphs (b), (c), and (d) depict gated Ly6G-Ly6C^+^ (d), Ly6G^+^Ly6C^−^ (c), and Ly6G^+^Ly6C^+^ (e) double positive cells. Backgating confirmed that all three populations were CD11b^+^. (e) Time course of the absolute number of viable cells in spleens of NOD and C5BL/6 mice. Data presented as mean ± SEM (*n* = 8–12 spleens). ^*∗∗*^ *p* < 0.05. ^*∗∗∗*^ *p* < 0.001.

To test our hypothesis that neonatal NOD mice are altered in their development of the immune system, we stained splenocytes from 1–7-day-old NOD and C57BL/6 mice with the CD11b, Ly6G, and Ly6C specific antibodies. Splenocytes from the two strains had significantly different proportions of CD11b^+^ cells at PND 1 (Figure 1(a)). Furthermore, they exhibited highly distinct expression profiles of the two myeloid lineage markers (Figures 1(b)–1(d)). Splenocytes from NOD mice held a high and increasing proportion of CD11b^+^Ly6G^+^Ly6C^−^ cells from PND 1–7 (Figure 1(b)), a minor proportion of CD11b^+^Ly6G^−^Ly6C^+^ cells (Figure 1(c)), and very little CD11b^+^Ly6G^+^Ly6C^+^ cells (Figure 1(d)). In contrast, splenocytes from C57BL/6 mice displayed a high proportion of CD11b^+^Ly6G^+^Ly6C^+^ cells, an increasing proportion of CD11b^+^Ly6G^−^Ly6C^+^ cells, and almost no CD11b^+^Ly6G^+^ Ly6C^−^ cells (Figures 1(b)–1(d)).

### 3.2. Livers of Newborn NOD Mice Are Rich in Cells with a Granulocytic Phenotype

**Figure 2:**
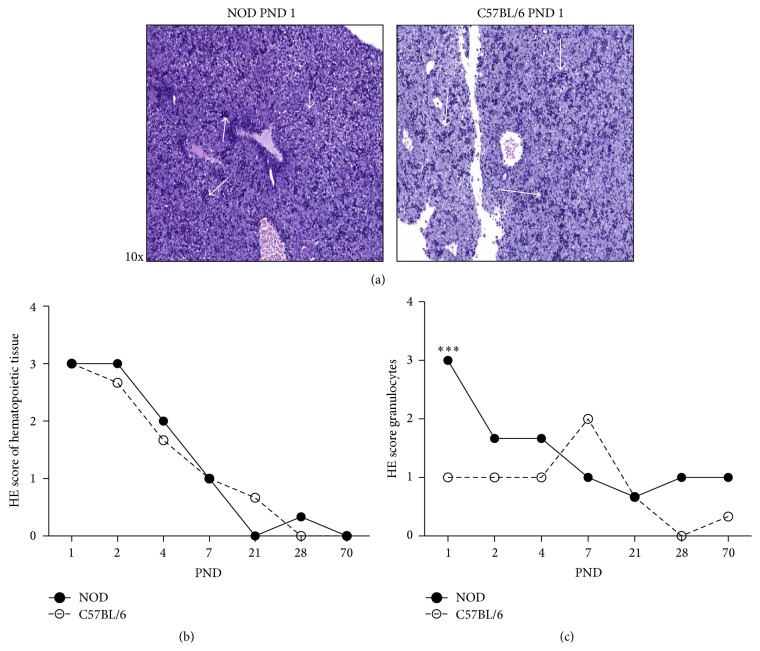
The presence of granulocytic myeloid derived cells is high in NOD mice livers. (a) Hematoxylin and eosin (HE) staining of neonatal PND 1 liver tissue sections from NOD and C57BL/6 mice. Arrows indicate hematopoietic tissue in the liver of newborn mice at PND 1. 10x magnification. (b) HE scores of hematopoietic tissue at PND 1–70 in NOD and C57BL/6 mice. Two persons evaluated HE score in a blinded fashion (*n* = 3). (c) HE score of granulocytes at PND 1–70. ^*∗∗∗*^ *p* < 0.001.

The vast difference in the myeloid differentiation patterns in spleens from newborn NOD and C57BL/6 mice prompted us to inspect the hematopoietic tissue in the livers of the newborn mice as well as characterizing the phenotype of the cells present. Sections of haematoxylin and eosin (HE) stained livers were visually examined with regard to the number, type, and localization of myeloid derived differentiating cells (Figure 2(a)). No difference in the amount of hematopoietic tissue was observed (Figure 2(b)) and no difference was observed in the rate of differentiation (Figure 2(c)). Assessment of the number of differentiated cells in the liver sections demonstrated that, compared to C57BL/6 mice, NOD mice had a significant higher level of cells with a polylobed or ringed nuclei at PND 1, 2, and 4 in the liver (Figure 2(c)). These cells were present in the liver as far as to PND 70 in NOD mice, though in much lower numbers than seen in the newborn mice.

**Figure 3:**
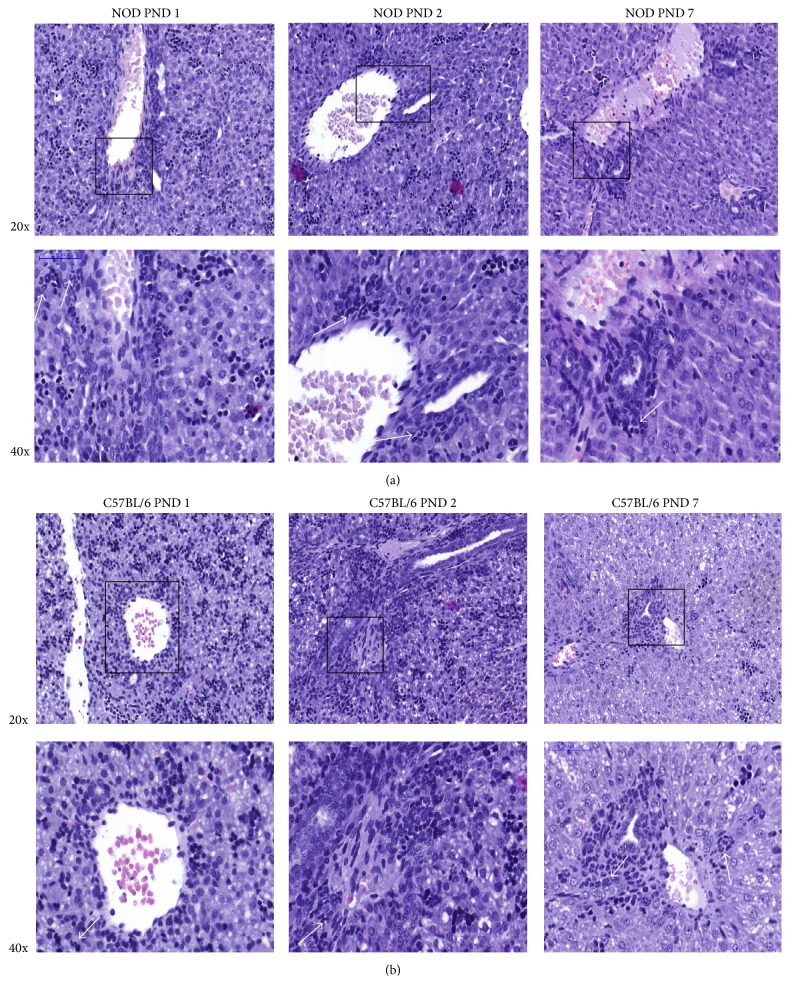
In NOD mice the granulocytic phenotype of cells accumulated around the hepatic portal veins. Hematoxylin and eosin (HE) staining of neonatal PND 1–7 liver tissue sections from NOD (a) and C57BL/6 mice (b).

Interestingly, in NOD mice the granulocytic phenotype of cells accumulated around the hepatic portal veins at PND 1 (Figure 3(a)), while in the C57BL/6 mice these cells were fewer and scattered in the tissue of the liver, with a gradient towards the blood vessels (Figure 3(b)). While the two mouse strains had similar numbers of hematopoietic stem cells in the livers at PND 1, they differed greatly in the number and in the localization of the differentiating granulocytic cells.

### 3.3. More Immature Granulocytes in the Spleen of NOD Mice Compared to C57BL/6 Mice

Visual examination of neonatal liver and spleen revealed a large proportion of granulocytic cells in different differentiation stages in NOD mice compared to C57BL/6 mice. To further assess their differentiation we measured the expression of* Elane* (neutrophil elastase),* Arg1* (arginase 1), and* Hp* (haptoglobin), all genes expressed in various differentiation steps in granulocytes [34–36].

**Figure 4:**
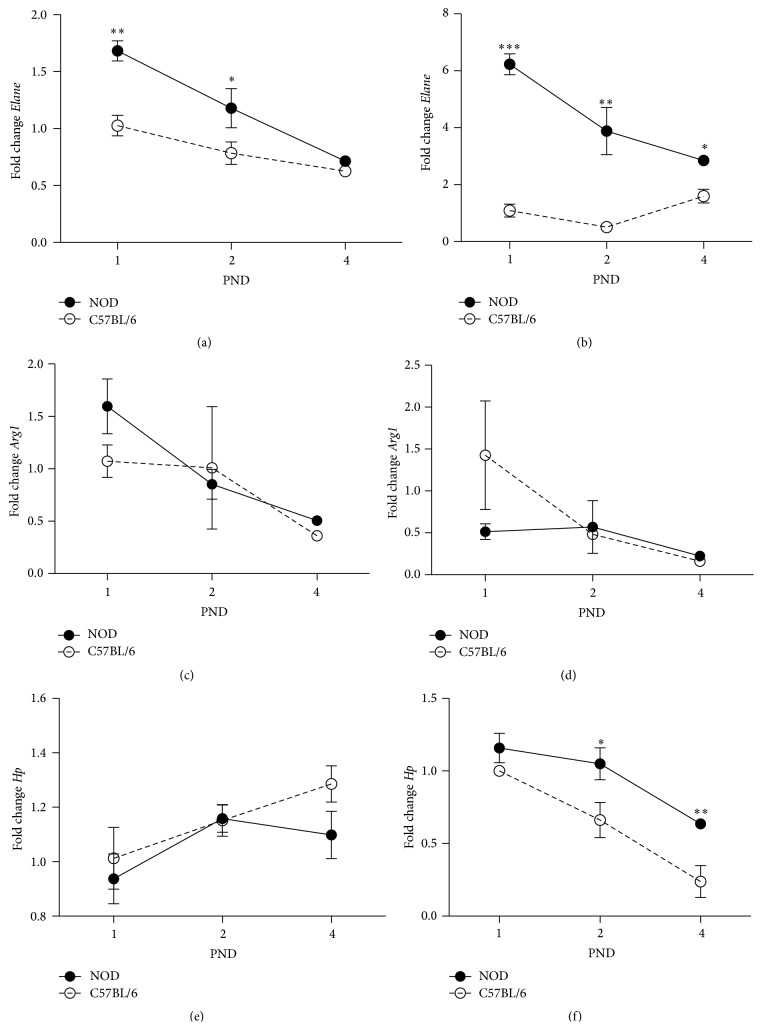
Relative gene expression of genes expressed in differentiation of myeloid cells in liver and spleen at PND 1, 2, and 4 of NOD and C57BL/6 mice. Data were normalized to* Actb* and then to C57BL/6 PND 1, which were defined as value 1. All samples were quantified in triplicate. All groups were included in a 2-way ANOVA. ^*∗∗∗*^ *p* < 0.001,  *p* < 0.05. (a) Expression of* Elane* encoding elastase in liver. (b) Expression of* Elane* encoding elastase in spleen. (c) Expression of* Arg1* encoding arginase in liver. (d) Expression of* Arg1* encoding arginase in spleen. (e) Expression of* Hp* encoding haptoglobin in liver. (f) Expression of* Hp* encoding haptoglobin in spleen.

In both liver and spleen,* Elane expression* was significantly higher in NOD mice at PND 1-2 and PND 1–4, compared to C57BL/6 mice (Figures 4(a) and 3(b)).* Arg1* expression was similar between the two strains at PND 1–4; however,* Arg1* was 200x higher expressed in liver compared to spleen. In spleen at days 2 and 4,* Hp* expression was higher in NOD mice (Figure 4(f)). No difference was observed in liver (Figure 4(e)), but* Hp* was expressed 50x higher in spleen than in liver, indicating that the final maturation of granulocytes takes place outside the liver.

**Figure 5:**
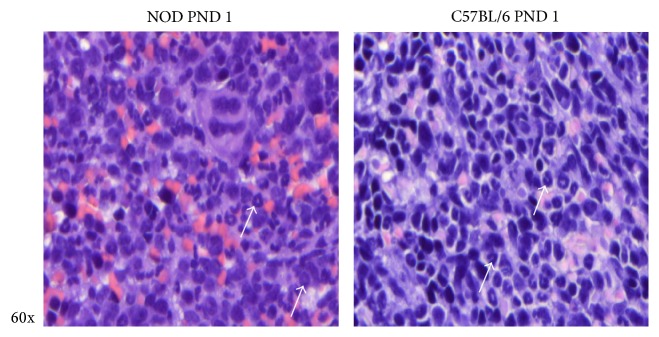
Hematoxylin and eosin (HE) staining of neonatal spleen PND 1 from NOD mice and C57BL/6. Arrows depict granulocytes at different stage of granulopoiesis. 60x magnification.

Visual examination of spleen sections revealed that splenocytes from NOD mice had a tendency to ring-shaped rather than polylobed morphology, indicating presence of granulopoietic stage 1 myeloblasts. C57BL/6 mice had a population of cells displaying the more polylobed morphology, of more mature neutrophils (Figure 5), indicating that relatively more immature granulocytes are present in the spleen and liver of NOD mice.

### 3.4. Bone Marrow-Derived HSCs from NOD Mice Stimulated with Bacteria Are Able to Differentiate into Ly6C^+^ Cells

**Figure 6:**
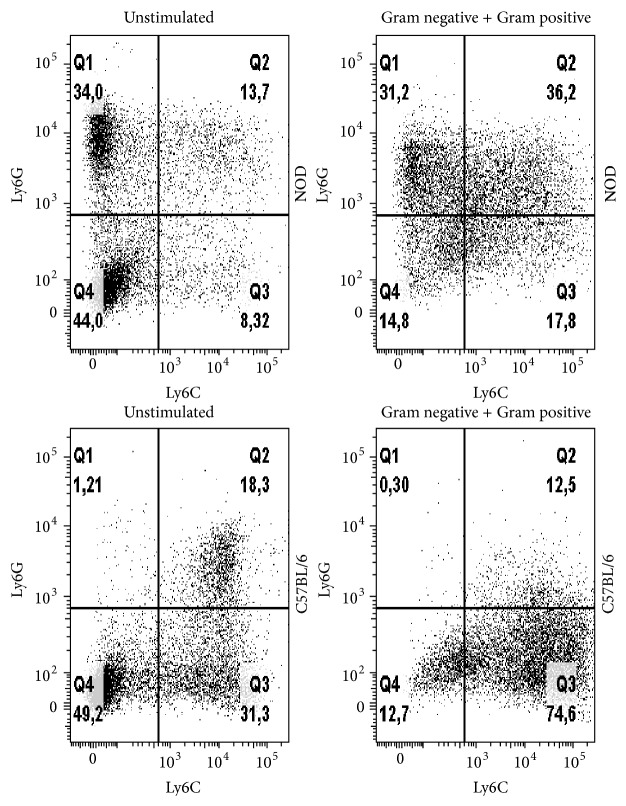
Bacterial stimulation of cultured bone marrow cells from NOD and C57BL/6 mice. On day 3 of* in vitro* culturing of bone marrow cells from NOD and C57BL/6 mice, mix of cells was stimulated as Gram-positive and Gram-negative bacteria (each MOI 0,5). On day 6 the cultured cells were harvested and analysed by flow cytometry. All CD11b^+^ cells were defined positive or negative for Ly6C and Ly6G. Plots are representative for 1 of 4 experiments.

A deficiency in the coding region of Ly6C has previously been described in NOD mice [26], which may explain the low Ly6C cell profile observed in the spleen of the NOD mice. To test if bacterial stimulation of the HSCs initiates the expression of more Ly6C^+^ cells, we stimulated bone marrow cells from adult NOD and C57BL/6 mice with a mixture of Gram-positive and Gram-negative bacteria and measured the expression of Ly6G and Ly6C (Figure 6). In nonstimulated cells, cells from C57BL/6 mice expressed 18.3% CD11b^+^Ly6G^+^Ly6C^+^ cells and 31.3% CD11b^+^Ly6G^−^Ly6C^+^ cells and almost no CD11b^+^Ly6G^+^Ly6C^−^ cells. In contrast and very similar to our findings in spleen cells from new born mice, bone marrow cells from NOD mice exhibited a high proportion of Ly6G^+^ cells 34.0% and 8.3% Ly6C^+^. When stimulated with bacteria, bone marrow cells from NOD mice developed a higher proportion of cells CD11b^+^Ly6G^−^Ly6C^+^ (17.8%) of which 36.2% were CD11b^+^Ly6G^+^Ly6C^+^. For comparison, cells from C57BL/6 mice changed from expressing both CD11b^+^Ly6G^+^Ly6C^+^ and CD11b^+^Ly6G^−^Ly6C^+^ cells to almost exclusively expressing CD11b^+^Ly6G^−^Ly6C^+^ cells.

These results indicate that bacterial stimulation may overcome the genetic predisposition, affecting the expression of Ly6C.

### 3.5. Higher Expression of* Reg3g* in the Intestine of Neonatal NOD Mice Is Associated with a Restriction of Surface Attachment of Gram-Positive Bacteria and a Low Expression of Muc2

**Figure 7:**
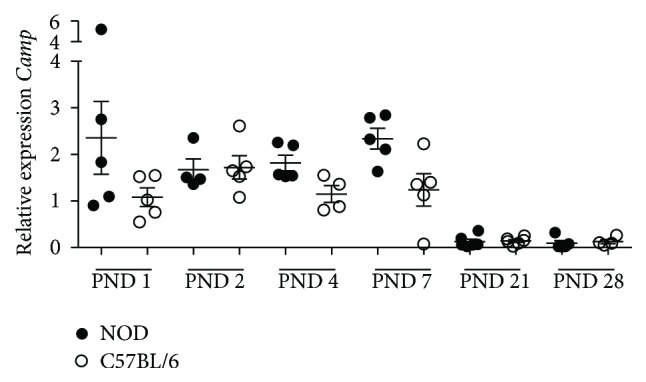
Relative gene expression of* Camp*, analyzed in intestine tissue from NOD and C57BL/6.

**Figure 8:**
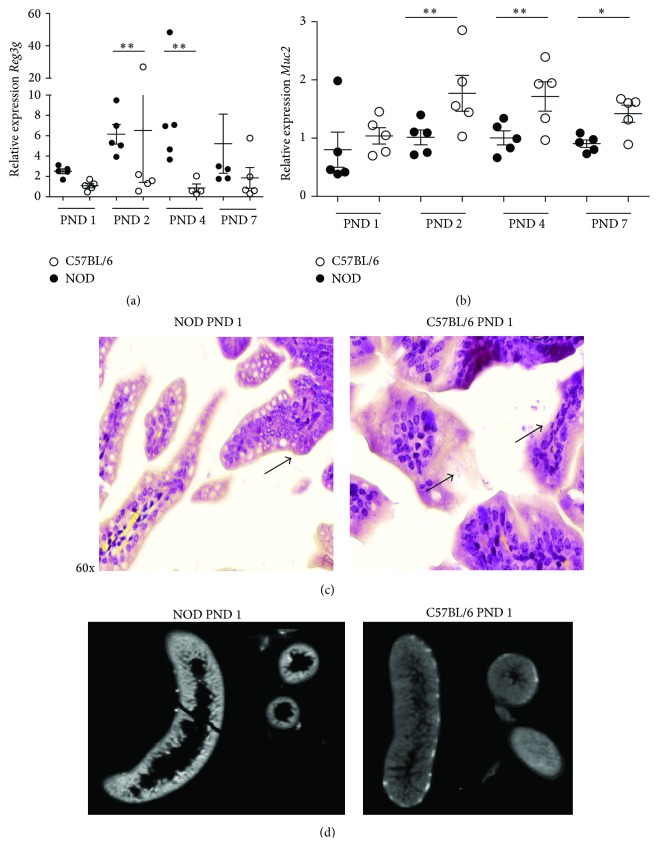
Relative gene expression of* RegIIIγ* analyzed in intestine tissue from NOD and C57BL/6 mice correlates with Gram-stain sections of the same intestines and OPT scanning. Data were normalized to* Actb* and then to C57BL/6 PND 1, which were defined as value 1. All groups were included in a 2-way ANOVA. ^*∗∗*^ *p* < 0.05. (a) Relative gene expression of* Reg3g*, analyzed in intestine tissue from NOD and C57BL/6. (b) Relative gene expression of* muc2* in intestinal tissue from NOD and C57BL/6. (c) Gram-stain sections of intestine 60x magnification. Arrows indicate presence of bacteria. In sections of NOD intestine (left side) bacteria were present in the lumen but very few and mostly Gram-positive bacteria were attaching to the villi. In contrast a larger amount of both Gram-negative and Gram-positive bacteria was attaching to the surface of the villi in the intestine of C57BL/6 mice. (d) PND 1 mouse intestine autofluorescence image acquired with the OPT system. Digital sections of the PND 1 intestine illustrated in gray-scale demonstrating autofluorescence anatomy data in NOD and C57BL/6. Intestinal sections from C57BL/6 mice displayed more dens villi compared to NOD mice.

The production of the antimicrobial cathelicidin-related antimicrobial peptide (*Camp*) and Reg3g in the neonatal intestine changes during maturation [37].* Camp* was equally expressed by the two strains and only before weaning (Figure 7). In contrast, Reg3g that normally is weakly produced until weaning [38] had increased expression in NOD mice at PND 2–7 compared to C57BL/6 (Figure 8(a)). Upregulation of* Reg3g* has been associated with an aberrant production of the secreted mucus protein Muc2 [39]. Hence, we measured the expression of* Muc2* in the neonatal period and found that* Muc2* was significantly lower from PND 2–7 in intestine of NOD mice (Figure 8(b)). Reg3g is proposed to primarily target Gram-positive bacteria [18] and next we examined Gram-stained sections of the intestine at PND 1–7. Presence of both Gram-positive and Gram-negative bacteria was observed in the lumen of both NOD and C57BL/6 mice (Figure 8(c)) However, a limited number of bacteria were observed close to the mucosal surface of villi in the NOD intestine and those present were mostly Gram-negative. In comparison, the C57BL/6 intestine had both Gram-positive and Gram-negative bacteria associated with the mucosal surface (Figure 8(c)).

Finally, intestinal morphology between C57BL/6 and NOD mice differed between the strains, as the intestinal villi of C57BL/6 showed a more dense and thick structure compared to NOD mice (Figure 8(d)).

### 3.6. Higher Abundance of* Staphylococcus* in NOD Mice Compared to C56BL/6 Mice

To assess the gut microbiota at PND 1–7 we analyzed samples of the entire intestine of C57BL/6 and NOD mice by tag-encoded 16S rRNA gene amplicon high throughput sequencing (6,238,023 fully overlapping raw reads, 5,575,454 after chimera purging). Four samples with less than 50,000 reads were excluded from further analysis. Remaining samples yielded on average 179,081 reads (STD +/−).

**Figure 9:**
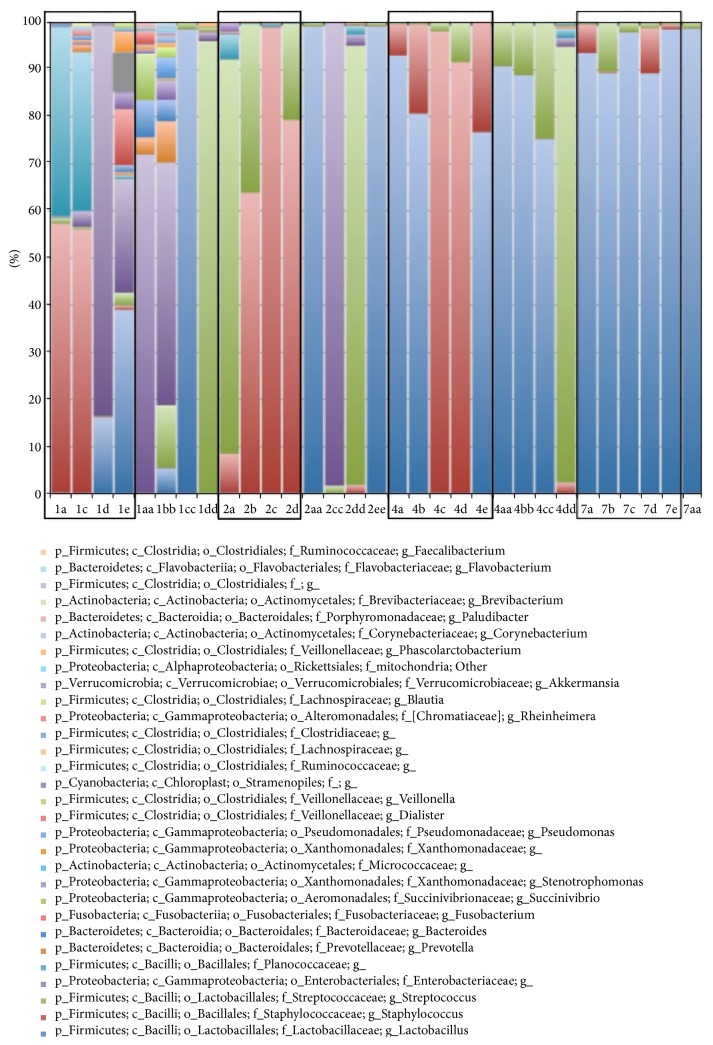
Microbiota composition of the intact intestine from C57BL/6 and NOD mice at PND 1, 2, and 4 as determined by 16S rRNA gene amplicon high throughput sequencing.* Brackets indicate phylum*.* Red*: Staphylococcus* (g)* (Firmicutes).* Blue*: Lactobacillus* (g)* (Firmicutes).* Purple*: Enterobacteriaceae* (f)* (Proteobacteria).* Green*: Streptococcus* (g)* (Firmicutes).* Turquoise*: Planococcaceae* (f)* (Firmicutes).* Black boxes indicate NOD mice*.

**Figure 10:**
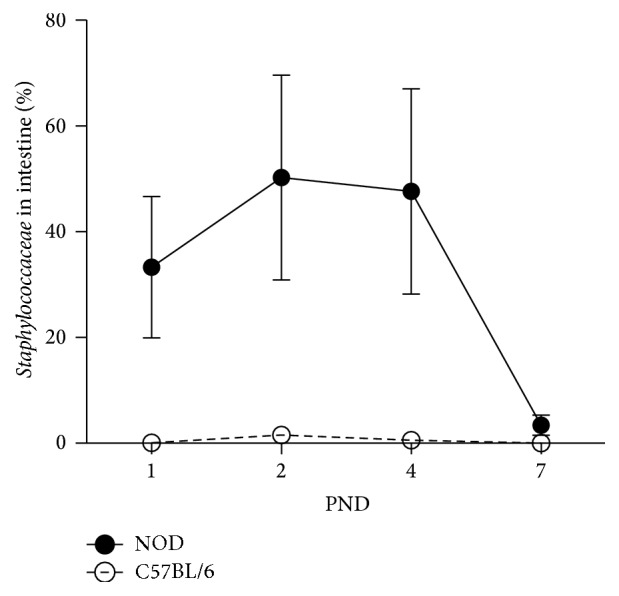
Microbiota composition of the intact intestine from C57BL/6 and NOD mice at PND 1, 2, and 4 as determined by 16S rRNA gene amplicon high throughput sequencing. Presence of* Staphylococcus* in the intestine of NOD and C57BL/6 mice. Data are expressed as percentage of bacteria present in intestine. Data are mean ± SEM (*n* = 4-5 intestines).

The number of observed bacterial species did not differ significantly between NOD and C57BL/6 mice (observed species NOD = 27.41 ± 12.33, C57BL/6 = 34.67 ± 20.17, *t* = −1.2, *p* = 0.26). ANOSIM analysis of uniFrac-based distance metrics revealed that the GM of the 2 mice strains did not differ significantly (unweighted *R* = 0.053, *p* = 0.163, weighted *R* = 0.077, *p* = 0.094).  Figure 9 shows the relative distribution of bacterial genera on PND 1, 2, 4, and 7 of NOD and C57BL/6 mice. The relative abundance of* Staphylococcus* was found to be significantly higher in NOD mice compared to C57BL/6 (*p*(Bonferroni-corrected) = 0.030) (Figure 10).

## 4. Discussion

Despite increased knowledge on the pathogenesis of type 1 diabetes and the role of the immune response in the development of type 1 diabetes in NOD mice, the early stages of the disease pathology remain poorly understood. Far the most studies are done in mice three weeks of age or older, in which the focus often is on the role of T cells infiltrating the pancreas and connecting lymph nodes [40, 41]. The events taking place prior to and causing this infiltration are largely unknown. Activation and action of some cells of the myeloid lineage are expected to be involved in the activation and polarization of T cells and also in recruitment of the T cells to the pancreas. Consequently, it is likely that hematopoietic events involving myeloid cells and taking place in the very early postnatal period are essential for the development. The key role of the GM in the development of type 1 diabetes in NOD mice has been firmly established [42]. NOD mice housed under germ-free conditions develop type 1 diabetes while mice reared under conventional conditions were demonstrated to be partly protected from the disease [43, 44] and GM manipulation through antibiotics or specific diets have been found to lower the incidence of autoimmune diabetes [6, 7]. The microbial influence on development and maturation of neonate immunity is however still only sparsely described but is likely to play a key role in the development of type 1 diabetes. Others have provided evidence that changes in the maternal microbiota during pregnancy and delivery influence the development of the immune system in NOD mice [40] and CS has been associated with increased type 1 diabetes incidence in humans [13]. Furthermore, gut bacteria were recently shown to be involved in regulation of hematopoiesis [45].

We here show that the hematopoiesis and the GM and the gut epithelium deviate in the NOD mice compared to C57BL/6 mice in the very first postnatal period. The neonatal expression of* Reg3g* and* Muc2* in the gut is deviating in NOD mice and this coincides with vast differences in the GM of NOD and C57BL/6 mice and is perhaps most interesting, in the number of bacteria associated with the mucosal surface. In parallel with these findings, we show that the proportions of Ly6C^+^ and Ly6G^+^ cells in the spleen of neonatal NOD mice deviate vastly from that of C57BL/6 mice, as the Ly6G^+^ cells dominate and Ly6C^+^ cell number is very low in NOD mice, but this can, at least* in vitro*, partly be circumvented by bacterial stimulation.

At birth the spleen and the liver constitute major hematopoietic tissues in mice and may represent important sites for encounters between microorganisms and immune system. Puga et al. found granulocytic infiltration of peri-marginal-zones of the spleen after postnatal mucosal colonization by microbes, showed the presence of bacterial 16s ribosomal RNA in these areas in the spleen, and found that the granulocytic phenotype was dependent on the microbiota [46]. Hence, the presence of bacteria or microbial components in the spleen (and liver) may influence the hematopoiesis here.

In the present study we demonstrate that spleen cells at PND 1–7 in NOD mice comprise a large population of immature granulocytic cells expressing CD11b^+^Ly6G^+^Ly6C^−^, while in C57BL/6 the far largest proportion of CD11b^+^ cells were Ly6C^+^Ly6G^+^ cells.

The lack of Ly6C expression in the bone marrow cells of NOD mice is long known [23, 26] and has been suggested to influence the propensity to development of type 1 diabetes, but the mechanism behind how these deviating myeloid cells influence disease development is largely unknown.* In vitro* we showed that bone marrow cells from NOD mice exhibit a similar Ly6C^−^ cell profile as the spleen cells of newborn mice, but these could be prompted to change to Ly6C expressing cells by the addition of bacteria. Thus, the presence of bacteria or microbial epitopes may have larger influence on a proper immune development in NOD mice compared to other, nondiabetic mouse strains.

Our microscopy data from NOD mice liver showed a larger number of granulocytic cells than in livers of C57BL/6 mice and abundant granulocyte like cells accumulated around the hepatic veins although the same amount of hematopoietic tissue present in NOD and C57BL/6 mice from PND 1. This might indicate either an earlier efflux of granulocytes from livers of C57BL/6 or a larger number of granulocytes are formed in the NOD mice. A postnatal efflux of hematopoietic cells from liver to spleen has previously been demonstrated in mice [47] and we showed here that in spleen granulocytic cells in early (*Elane* expressing) as well as late (*Hp* expressing) differential state are present while, in liver, primarily early differential state granulocytes are present, supporting the possibility that many immature granulocytes migrate from liver to spleen. Also, in spleen both* Elane* and* Hp* were more highly expressed in NOD mice and in the liver only* Elane* was higher expressed furthermore indicative of more granulocytic cells in these organs in NOD mice.

It is well established that, during the first postnatal weeks, Paneth cells are absent in the crypts of the mouse intestine and in this period the intestinal epithelium expresses* Camp* [37]. As expected, the expression of* Camp* decreased around the time of weaning and, at this time (PND 28), mature Paneth cells producing Reg3g are present. However, other cells such as goblet cells and enterocytes have also been demonstrated to express Reg3g [48] and are likely producers of this defensin at this early stage. In contrast, the expression of* Muc2* was significantly lower in NOD mice compared to C57BL/6 mice. We have earlier shown that the presence of a full GM in the postnatal period leads to upregulation in the gene transcription of* Muc2* [49]. In the present study we found that a larger number of bacteria were associated with the mucosa of C57BL/6 mice compared to NOD mice, which could be speculated to be the cause of the increased Muc2 expression. A lower production of* Muc2* would likely influence the composition of the microbiota, as glycans of mucin functions as source of nutrients for many bacteria [50]. Paassen et al. [48] have recently demonstrated that absence of* Muc2* was followed by upregulation of* Reg3g* in the intestine of muc2^−/−^ mice. Thus, the low expression of* Muc2* could explain the upregulated level of* Reg3g* in the NOD mice. When inspecting the morphology of the intestine of the newborn mice by OPT scanning, we observed considerable morphological differences between PND 1 NOD and C57BL/6 mice, as NOD mice intestine showed a thinner and less dense structure. Hence, physiological differences as well as differences in gene expression which may affect translocation of bacteria or microbial components from the gut were seen.

Reg3g binds peptidoglycan [18], expressed on the surface of Gram-positive bacteria. Our Gram-staining analysis revealed both Gram-negative and Gram-positive bacteria in the lumen of both mice strains. While both classes of bacteria were attached to the surface of the villi in the C57BL/6 mice, few Gram-negative bacteria were attached in NOD mice intestine. Interestingly, a bacteria profile enriched in* Staphylococcus* was prevailing in the intestine of NOD mice at PND 1–4. This genus of Gram-positive bacteria has previously been associated with children delivered by caesarean section and with an increased risk of developing type 1 diabetes [51]. The very different and less diverse bacterial composition found in newborn NOD mice is likely to influence gene expression in the gut and thus factors such as the mucus thickness. Hildebrand et al. reported that genetics directs 20% of the GM composition [52] and genetics in the gut epithelium may also be involved in our study, as the animals were reared in the same animal facility and handled by the same persons, giving different basis for colonisation of bacteria. Interestingly, the high level of* Staphylococci* found at PND 1–4 in NOD mice decreased dramatically at PND 7, which is in correspondence with the expression level of* Reg3g*. Whether* Staphylococci* are directly involved in the expression of* Reg3g* is however purely speculative.

In summary, we here demonstrate huge differences in the hematopoiesis, in GM, and in gut epithelium between NOD and C57BL/6 mice during the very first postnatal period. We propose that these differences might be involved in the predisposition for developing type 1 diabetes in the NOD mice.

## Highlights

- Hematopoiesis deviates in the NOD mice compared to C57BL/6 mice in the postnatal period.
- Neonatal expression of* Reg3g* and* Muc2* in the gut is deviating in NOD mice.
- Fewer bacteria attach to the intestinal surface in neonatal NOD.
- *In vitro microbial* stimulation of bone marrow cells leads to changes in hematopoiesis.

## Figures and Tables

**Table 1:** Gene expression assays used for qPCR.

Gene	Gene name	Assay ID
*Actb*	*Actin, beta*	Mn00607939_s1
*Elane*	*Elastase, neutrophil expressed*	Mm01168928_g1
*Arg1*	*Arginase*	Mm00475988_m1
*Hp*	*Haptoglobin*	Mn00516884_m1
*Camp*	*Cathelicidin antimicrobial peptide*	Mm00438285_m1
*Reg3g*	*Regenerating islet-derived 3 gamma*	Mm00441127_m1
*Muc2*	*Mucin 2*	Mm01276696_m1
